# The Development and Biomechanical Analysis of an Allograft Interference Screw for Anterior Cruciate Ligament Reconstruction

**DOI:** 10.3390/bioengineering10101174

**Published:** 2023-10-09

**Authors:** Sebastian Lifka, Yannik Rehberger, Klaus Pastl, Alexander Rofner-Moretti, Markus Reichkendler, Werner Baumgartner

**Affiliations:** 1 Institute of Biomedical Mechatronics, Johannes Kepler University Linz, 4040 Linz, Austria; werner.baumgartner@jku.at; 2 Surgebright GmbH, 4040 Lichtenberg, Austria; yannik.rehberger@surgebright.com (Y.R.); klaus.pastl@surgebright.com (K.P.); 3 Department of Orthopedic and Trauma Surgery, District Hospital Schwaz, 6130 Schwaz, Austria; alexander.rofner-moretti@kh-schwaz.at (A.R.-M.); markus.reichkendler@kh-schwaz.at (M.R.)

**Keywords:** anterior cruciate ligament (ACL) reconstruction, interference screw, allograft, finite element analysis (FEA), orthopedic implant, bovine model

## Abstract

Graft fixation during cruciate ligament reconstruction using interference screws is a common and frequently used surgical technique. These interference screws are usually made of metal or bioabsorbable materials. This paper describes the development of an allograft interference screw from cortical human bone. During the design of the screw, particular attention was paid to the choice of the screw drive and the screw shape, as well as the thread shape. Based on these parameters, a prototype was designed and manufactured. Subsequently, the first biomechanical tests using a bovine model were performed. The test procedure comprised a torsion test to determine the ultimate failure torque of the screw and the insertion torque during graft fixation, as well as a pull-out test to asses the ultimate failure load of the graft fixation. The results of the biomechanical analysis showed that the mean value of the ultimate failure torque was 2633 N mm, whereas the mean occurring insertion torque during graft fixation was only 1125 N mm. The mean ultimate failure load of the graft fixation was approximately 235 N. The results of this work show a good overall performance of the allograft screw compared to conventional screws, and should serve as a starting point for further detailed investigations and studies.

## 1. Introduction

The rupture of the anterior cruciate ligament (ACL), one of the major knee-stabilizing ligaments that connects the tibia to the femur, is one of the most common knee injuries in individuals who engage in physical activities and is the result of rapid deceleration or hyperextension [1,2,3,4,5]. The resulting instability of the knee joint often prevents a return to pre-injury activity levels and, in addition, there is a risk of further injury such as a meniscus tear which, in turn, can lead to early-onset osteoarthritis in the knee joint [6,7,8]. In order to restore the full knee stability, operative treatments using tendon grafts (autograft or allograft tissue) for reconstruction of the ACL are highly common [3,9]. Thus, after ACL reconstruction, the patient is able to return to the preoperative activity level in most cases [10,11,12].

Typically, the ACL reconstruction involves the following steps: removal of the ruptured ACL, graft harvesting (if an autograft is used), graft preparation, drilling of tibial and femoral tunnels in which the graft is placed similar to the original ACL, and graft fixation [13]. For the ACL reconstruction, a variety of different techniques and graft fixation devices exist [14,15,16]. For example, these devices can be classified based on their underlying mechanisms, which are compression, expansion and suspension [13]. Typical examples of fixation devices producing a compression load to the longitudinal axis of the graft are interference screws or bone plugs. The cross pin (Rigidfix) system produces a bulging of the graft, and is therefore based on the expansion mechanism [17,18]. Fixation devices like buttons, Swing Bridges and Ligament Plates fix the graft by suspending the graft into the femoral tunnel [13].

In this work, the focus is on the interference screw as an implant for graft fixation within the ACL reconstruction. These interference screws can either be made of metal or bioabsorbable materials [19,20,21,22]. Metal screws, on the one hand, show a slightly higher over all strength compared to bioabsorbable screws [23] but, on the other hand, exhibit some disadvantages like distortion of magnetic resonance imaging (MRI), the risk of joint rupture and the need for hardware removal [23,24]. Interference screws made of bioabsorbable materials do not show these disadvantages but, due to the lower strength compared to metal screws, deformation of the screwdriver tip may lead to an increased insertion torque and, as a consequence, a failure of the screw during insertion [25].

An alternative to metal or bioabsorbable screws are screws made of allograft human cortical bone, like the Shark Screw^®^ (surgebright GmbH, Lichtenberg, Austria). These allograft bone screws are used for fixation in fracture, osteotomy, and arthrodesis and therefore make additional application of non-human materials dispensable. Furthermore, these allogenic screws exhibit superior osteoconductive properties, which means they are integrated in the recipient bone by the continuous bone remodeling process, and therefore are completely converted into autologous bone [26,27,28,29].

Therefore, the aim of this work was the development and the initial biomechanical analysis of an interference screw for ACL reconstruction made of allograft human cortical bone. For this purpose, a first prototype of the allograft interference screw was designed and manufactured. On the one hand, the focus was particularly on the design of the screw drive, since not every screw drive that is used in metal or bioabsorbable screws can be manufactured in human cortical bone [30] and, on the other hand, on the design of the screw shape, the screw dimensions and the thread shape. Subsequently, an initial biomechanical analysis of the screw prototype regarding the overall performance of the implant in terms of ultimate failure torque of the screw and ultimate failure load of the graft fixation was performed using a bovine model.

At this point, it is worth mentioning a possible main advantage in terms of secondary stability of the allograft screws. Within a short time, rehydration of the freeze-dried screw leads to significant swelling. The screw diameter is thus slightly increased, which leads to an increase in compression on the graft and, thus, possibly to an increase in the stability of the fixation. In addition, the stability of the fixation should be further enhanced over time by the onset of the bone remodeling process [26,27,28] and the resulting ingrowth of the graft. However, these effects could not be investigated with the bovine model used here; therefore, further research is needed.

## 2. Materials and Methods

### 2.1. Design of an Allograft Interference Screw for ACL Reconstruction

#### 2.1.1. Design of the Screw Drive

For an interference screw, there are various requirements that must be met by the screw drive. The screw drive must be stable enough to withstand the torque that occurs when fixing the graft. Furthermore, the screw drive must allow the screw to be fully countersunk, which means that the diameter of the insertion tool must be smaller than the outer diameter of the screw. At the same time, however, the screw drive has to be as simple as possible to manufacture.

Based on the above requirements, some conventional screw drives can already be excluded. A standard external hexagon head, which is also used in the Shark Screw^®^, where the wrench size is larger than the outer diameter of the screw, would be extremely suitable in terms of stability, but has the disadvantage that complete countersinking of the screws is not possible. A simple slot drive does not provide sufficient strength, and is also susceptible to tilting of the insertion tool, which could lead to unintentional damage to the implant. The internal hexagon socket would be ideally suited for this application on the one hand, and the internal hexalobular socket on the other. However, since these complex screw drives are very difficult to fabricate in cortical bone, as fabrication requires fragile micro-mills to achieve the small radii, they are of limited use for this application.

Following from the above considerations, three possible screw drives were shortlisted for further investigations. First, the so-called claw clutch, which is already used with the Shark Screw^®^; second, the so-called three-bore drive, which was recently described in a prior publication [30]; and third, a modified version of a conventional external hexagon head, at which the wrench size is smaller than the core diameter of the screw. All three drives allow the screw to be fully countersunk and permit easy manufacturing in cortical bone.

**Figure 1:**
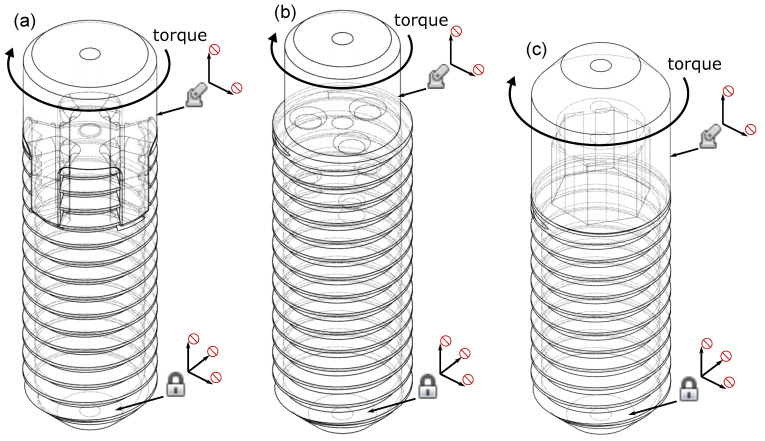
CAD models of the screws with claw clutch (**a**), three-bore drive (**b**), and modified external hexagon drive (**c**), with the respective insertion tool merged. This setup is the basis for the FEA; the torque was applied to the insertion tool in each case. Structural constraints were applied to both the insertion tool and the screw, and are shown schematically in each panel.

In order to make an estimation of whether the screw drives are able to withstand the torques occurring during graft fixation, a finite element analysis (FEA) using the software Fusion 360 (Autodesk, Inc.; San Rafael, CA, USA), similarly to a previous publication [30], was performed. All edges in the CAD design were given a small radius in order to avoid singularities due to sharp edges [31]. All threads of the screws were fully modeled to simulate the behavior as realistically as possible. The outer screw diameter used in the FEA is 8 mm for all simulated screw drives. The screws and the respective insertion tools were modeled as separate files, and then merged for the FEA leading to a contact problem (Figure 1). For the contact problem, the contact type “Separation” of Fusion 360 was used. All screw drives were modeled to form-fit and be as simple as possible, to reduce complexity and computational effort.

The FEA was designed as a static stress analysis with linear isotropic material properties in order to reduce the complexity of the problem and thus also processing power. Since the idea is to find the torque that the screw can just withstand (i.e., the yield strength should not be reached), we remain in the linear range of the stress–strain diagram. This approach is therefore a good approximation [30,32]. The materials chosen for the FEA were freeze-dried human cortical bone for the screws and stainless martensitic chromium steel 1.4034 for the insertion tools, respectively [30]. The detailed material properties can be found in Table 1.

Structural constraints were applied to both the screw and the insertion tool (schematically shown in Figure 1). The bottom face of the screw was fixed in all three axes, the cylindrical face of the insertion tool was pinned in radial and axial directions to prevent undesired movement, except for tangential movement due to the applied torque. The torque was applied in negative z-direction to the insertion tool as structural load. The contacts between the screw and the insertion tool were defined using the “automatic contacts” function with the default contact tolerance. The FEA used the Fusion 360 default mesh with linear tetrahedral elements. The average element size of the mesh was chosen between 1% and 10%, based on the model and scaled per component. No adaptive meshing was used to increase computational speed. Each trial was solved using the automatic “Cloud Solve” function of Fusion 360 [30].

The evaluation of the results of the FEA was carried out by considering the von Mises stress distribution [33] at certain loads. The peak values of the von Mises stresses were not taken into account, because they are unrealistic high due to singularities, predominantly at the contacts between screw and insertion tool and at edges with small radius. If these high-stress areas are only local, it is very likely that the screw will withstand the load anyway. If, on the other hand, the high von Mises stresses are distributed over a large area, the screw is likely to fail at this load and above [30].

However, the modified external hexagon drive slightly over performs compared to the other two drives. Due to the fact that an external hexagon is already used in existing Shark Screw^®^ systems, and experience is therefore available in production and in the admission process, the modified external hexagon was chosen as screw drive for the interference screw. The modified external hexagon head has a wrench size of 5 mm and a height of 3 mm, thus the diameter of the insertion tool can be designed smaller than the outer diameter of the screw (in this case 8 mm), which allows the screw to be fully countersunk.

#### 2.1.2. Design of the Screw Thread

Besides the screw drive, the thread is another very important part of an interference screw. On the one hand, the thread must allow fast and easy screwing and fixing, and on the other hand, the thread must not be sharp-edged, so as to prevent injury to the graft. Initial tests using regular metric threads, similar to those used for standard Shark Screws^®^, showed that the edges of this thread type are too sharp, leading to indefensible damages of the graft. Therefore, the thread-edges have to be designed in a more flattened way, in order to prevent injury to the graft. Initial experiments with metric threads also showed that screw insertion was slow and required many turns of the screw. Since time often plays a central role in ACL reconstructions, it is necessary to keep the operating time as short as possible. To enable the screw to be inserted as quickly as possible, it was decided to design the thread as a two-start thread. Each thread has a pitch of 3 mm, which in combination results in a pitch of 1.5 mm of the two threads to each other.

#### 2.1.3. Design of the Screw Shape and Screw Dimensions

The final step of the screw design involved the shape and overall dimensions of the allograft interference screw. Since the outer diameter of the screw is limited by the thickness of the cortex of the donor bone (femur/tibia) from which the allograft screws are made [34,35], the outer diameter of the screw was chosen to be 8 mm. This is a common diameter for interference screws [36] and can still be reasonably produced for allograft screws. The overall length of the allograft interference screw is limited to just over 20 mm, which is at the lower limit of conventional interference screw length, but quite common [37,38,39]. This is again due to the properties of the base material. The required thickness of the cortical bone (>8 mm) is only present over a certain length, which limits the overall length of the screw. To facilitate placement of the screw in the drill tunnel adjacent to the graft, the screw shape should be tapered at the opposite end to the screw head. To allow insertion of the screw using a guide, the screw has to be cannulated with a 1.25 mm through hole.

**Figure 2:**
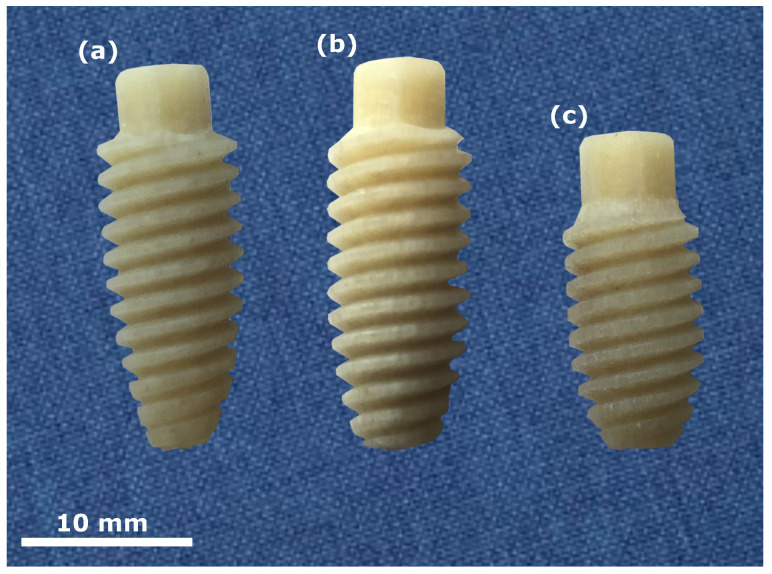
Three different prototype versions designed and manufactured by surgebright GmbH. All versions have an outer diameter of 8 mm, an external hexagon head with a wrench size of 5 mm and a height of 3 mm, a flattened double thread with a pitch of one thread of 3 mm (1.5 mm both threads in combination), a tapered outer shape, and a through hole with a diameter of 1.25 mm. The three versions differ in length and outer shape. Version (**a**) has an overall length of 21 mm, and is the most tapered one. Version (**b**) also has an overall length of 21 mm, but is slightly less tapered than version (**a**). Version (**c**) has an overall length of 18 mm, and is the shortest and the least tapered one.

**Figure A1:**
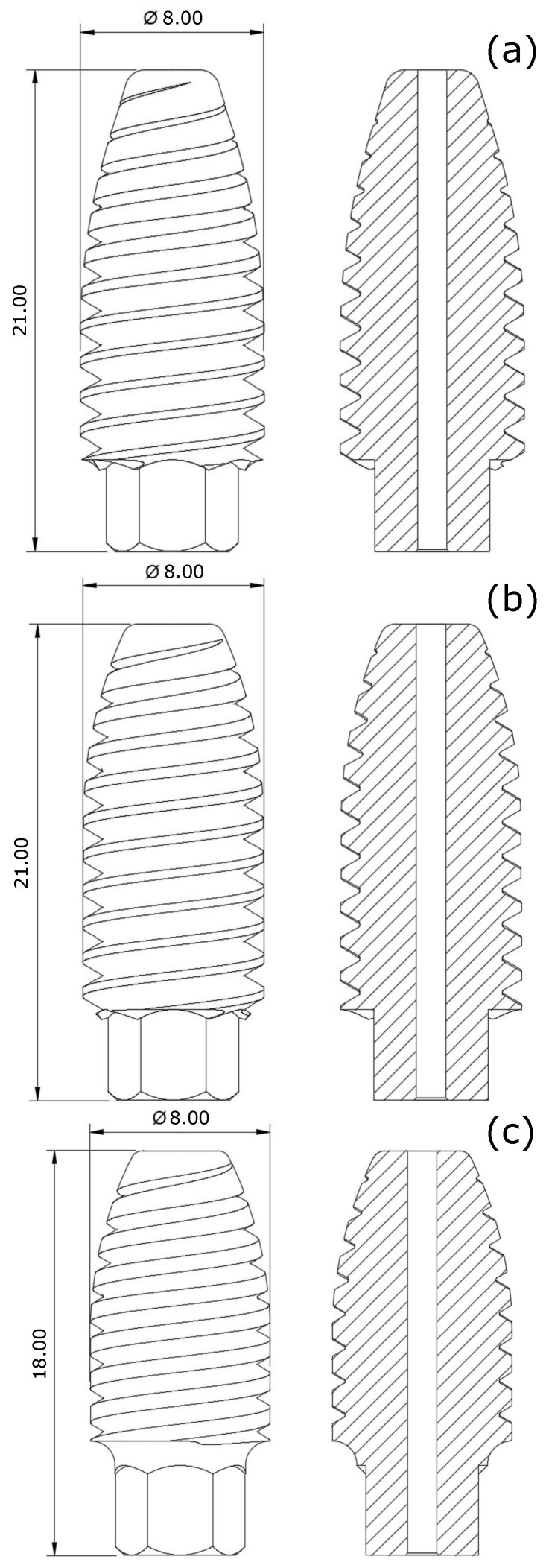
Technical drawings of prototype screw versions (**a**–**c**) from Figure 2.

Based on these considerations, three different versions ((a), (b), and (c)) of prototypes of the allograft interference screw shown in Figure 2 were designed and manufactured by surgebright GmbH. All versions have an outer diameter of 8 mm, an external hexagon head with a wrench size of 5 mm and a height of 3 mm, a flattened double thread with a pitch of one thread of 3 mm (1.5 mm both threads in combination), a tapered outer shape, and a through hole with a diameter of 1.25 mm. The three versions differ in length and outer shape. Version (a) has an overall length of 21 mm, and is the most tapered one. Version (b) also has an overall length of 21 mm, but is slightly less tapered than version (a). Version (c) has an overall length of 18 mm, and is the shortest and the least tapered one. Based on initial insertion tests in bovine cancellous bone performed by experienced orthopedists, all three versions were tested and compared. The behavior during screw insertion and thus graft fixation was similar for all three versions. The torque required was felt to be acceptable, and the speed of insertion was also quite fast due to the two-start thread. The graft itself showed no tendency to rotate with the screw in the tunnel, which is crucial for easy screw insertion. The visual condition was consistently very good in all three versions, and the flattened thread did not damage the grafts. However, the decision on which version to use for the biomechanical tests was made, and was version (a) in Figure 2. The decision was justified by the fact that a greater overall length of the screw should have advantages in the stability of fixation compared to version (c). Compared to version (b), the more tapered shape of version (a) allows easier and better placement of the screw in the drill channel. Figure A1 in Appendix A provides technical drawings of the various prototype versions shown in Figure 2.

### 2.2. Biomechanical Analysis Using a Bovine Model

#### 2.2.1. Graft Preparation

**Figure 3:**
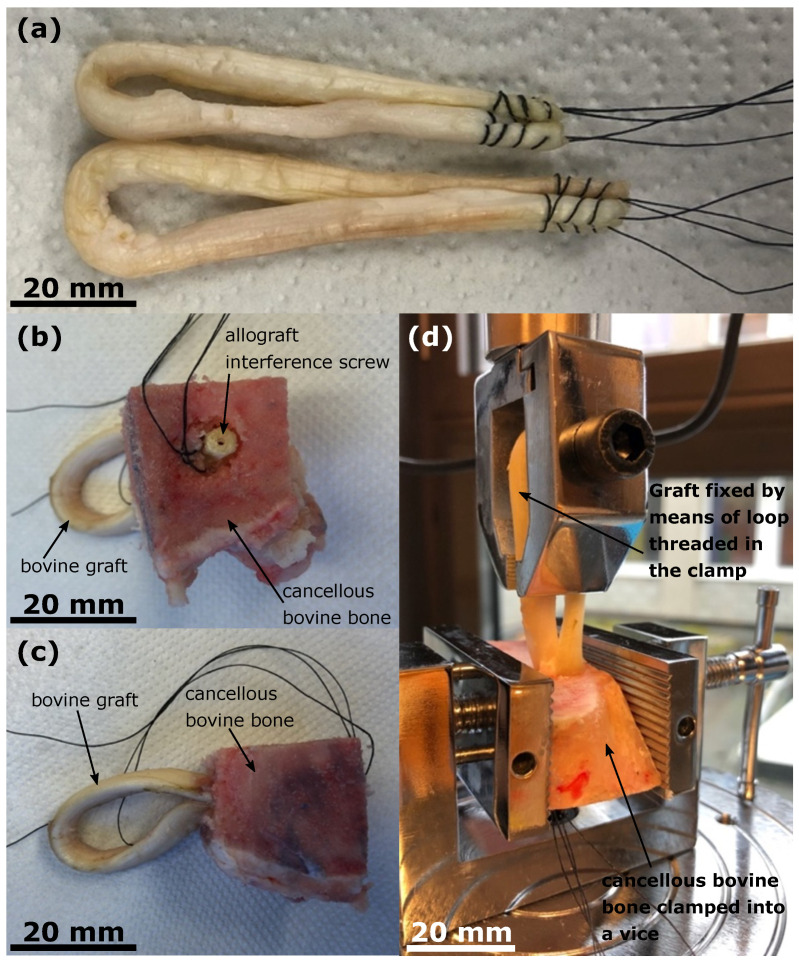
(**a**) Doubled bovine tendons with a length between 7 cm to 8 cm, diameters ranging from 7 mm to 9 mm, and stitched ends. (**b**,**c**) Bovine graft fixed with the allograft interference screw in a cancellous bovine block, top and side views. (**d**) Specimen fixed in a motorized force test bench. The bovine bone block was fixed in a vice connected to the movable table of the force test bench. The graft was attached to the stationary upper clamp, and connected to the load cell of the force test bench using the loop resulting from the single tendon fold.

Bovine tendons with a total length of about 14 cm to 16 cm were single-folded, and the ends were stitched together using a simple yarn. This resulted in doubled grafts with a length of 7 cm to 8 cm and diameters ranging from 7 mm to 9 mm (Figure 3a). Prior to the biomechanical tests, the bovine tendons were stored at −18 ${}^{\circ }C$.

#### 2.2.2. Graft Fixation

The prepared grafts were fixed at the stitched end in bovine cancellous bone blocks, which were stored at − 18 ${}^{\circ }C$ prior to the biomechanical tests. For this purpose, a through hole was pre-drilled through the bone block with a diameter ranging from 8 mm to 9.5 mm, depending on the graft diameter. The graft was then threaded through the bore tunnel and tensioned. The allograft interference screw was screwed into the drill tunnel next to the graft until it was fully countersunk (Figure 3b,c). During screw insertion, the maximum insertion torque was measured using an electronic torque meter (Wisretec BDA2-030; Wisret Precision Co., Ltd., Shenzen, China) and recorded.

#### 2.2.3. Measurement of the Ultimate Failure Load of the Graft Fixation

In order to measure the ultimate failure load of the graft fixation, a motorized force test bench PCE-MTS 500 (PCE Deutschland GmbH, Meschede, Germany) with an S-type load cell PCE-PFG 2K (PCE Deutschland GmbH, Meschede, Germany) was used. The cancellous bovine bone block in which the graft was fixed with the interference screw was clamped into a vice connected to the moving table of the force test bench. The graft was attached to the stationary upper clamp, connected to the load cell, of the force test bench using the loop resulting from the single tendon fold. For this purpose, the screw of the upper clamp was threaded through the loop of the graft. The screw was not tightened, which prevented the graft from jamming and possibly being damaged. After the specimen was installed properly (Figure 3d), the moving table of the force test bench was constantly moved downwards at a speed of 10 mm min^−1^ until failure of the graft fixation. At the same time, the tensile force acting on the graft was measured, and the maximum value at fixation failure was recorded. The tensile force was applied in the direction of the axis of the bore tunnel, so that the worst-case scenario was simulated.

#### 2.2.4. Measurement of the Ultimate Failure Torque

The ultimate failure torque was measured, as described in a previous publication [30], by clamping the interference screw at the thread in a three-jaw chuck of a lathe (PD 400, PROXXON S.A., Wecker, Germany) and then loading it with a torque until material failure. The ultimate failure torque was measured and recorded with an electronic torque meter (Wisretec BDA2-030; Wisret Precision Co., Ltd., Shenzen, China).

#### 2.2.5. Statistical Analysis

Independent-samples *t*-tests were performed to examine significant differences in results between 7 mm and 9 mm diameter grafts in terms of insertion torque and ultimate load. Previously, these parameters were tested for normal distribution within each group using the Shapiro–Wilk test. Additionally, further correlation analysis in terms of correlation between insertion torque and ultimate failure load for all graft diameters were performed. The level of significance was set to α=0.05 for all statistical tests. The statistical analysis was performed using Python3 and the Scipy package.

## 3. Results

### 3.1. Results of the FEA

**Figure 4:**
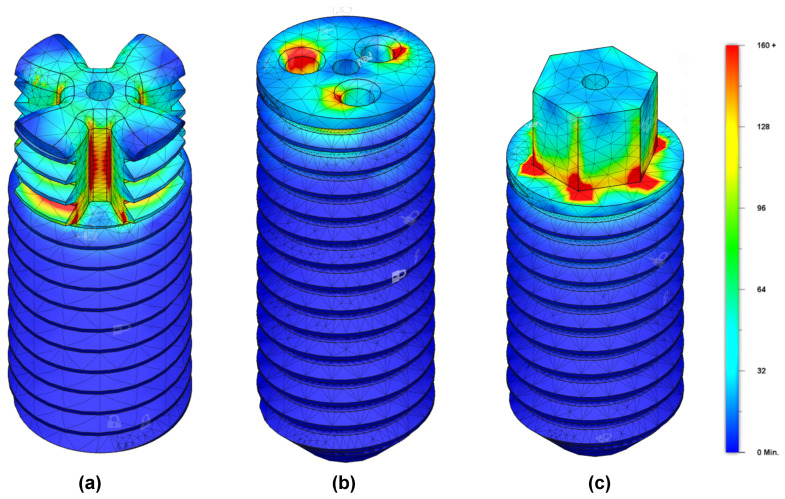
FEA of (**a**) a claw clutch screw drive, (**b**) a three-bore screw drive, and (**c**) a modified external hexagon screw drive. The simulated outer screw diameter is 8 mm, and is the same in all three panels. The torque applied to all screw drives is 2500 N mm. At this applied torque, the investigated screw drives are very likely to fail according to the FEA. The red zones represent areas with von Mises stress greater than 160 MPa and, therefore, areas with von Mises stress greater than the yield strength of cortical human bone (approx. 108 MPa [32]). The color bar is valid for all panels.

Figure 4 shows the results of the FEA for the above-described screw drives. The torque applied on all screw drives in Figure 4a–c is 2500 N mm. After trying different values for the applied torque, it can be concluded from Figure 4 that at the applied torque, the investigated screw drives are very likely to fail, according to the FEA. So one can conclude that all three drives are roughly in the same order of magnitude regarding ultimate failure torque.

### 3.2. Results of the Biomechanical Analyses

In total, n=12 different specimens of the allograft interference screw were analyzed (n=8 for the 7/7.5 mm graft diameter group and n=4 for the 9 mm graft diameter group). In order not to destroy all allograft interference screws, the ultimate failure torque analysis was performed only for n=3 specimens. The results of the biomechanical test are listed in Table 2.

**Figure 5:**
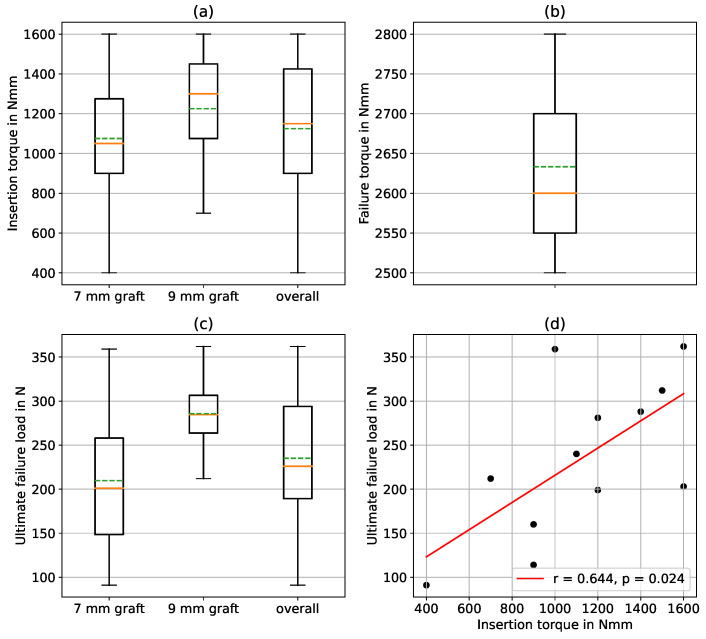
Results of the biomechanical analyses. (**a**) Box plot showing the median values (solid orange line), the mean values (dashed green line), and quartiles of the maximal insertion torques during graft fixation for the 7 mm graft (n=8), the 9 mm graft (n=4), and for all together (n=12). (**b**) Box plot showing the median value (solid orange line), the mean value (dashed green line), and quartile of the ultimate failure torque of the screw (n=3). (**c**) Box plot showing the median values (solid orange line), the mean values (dashed green line), and quartiles of the ultimate failure load of the graft fixation for the 7 mm graft (n=8), the 9 mm graft (n=4), and for all together (n=12). (**d**) Correlation plot between the insertion torque during graft fixation and the ultimate failure load of the graft fixation for all graft diameters (r=0.644, p=0.024, α=0.05, n=12).

The mean value for the maximum insertion torque of the 7 mm graft group was 1075±377.02 N mm and 1225±386.22 N mm for the 9 mm graft group, thus showing no significant difference (p=0.533). The results for the ultimate failure load of the graft fixation showed mean values of 209.75±92.24 N and 285.75±61.32 N for the 7 mm and the 9 mm graft group, respectively, again showing no significant difference (p=0.171). The failure mode in all specimens was graft and screw pull-out. The ultimate failure torque test showed that the maximum torque that the screw can withstand on average is with 2633.33±152.75 N mm, more than twice as high as the mean insertion torque during graft fixation. The failure mode in all specimens was a fracture in the area of the screw head. Figure 5a–c show the presented results in Table 2 visualized as box plots with the median values (solid orange line), the mean values (dashed green lines), and the quartiles. Figure 5d shows the correlation plot between the insertion torque and the ultimate failure load of the graft fixation. A significant correlation between the two parameters could be observed (p=0.024).

## 4. Discussion

In this work, an allograft interference screw manufactured from cortical human bone was developed and biomechanically analyzed. During development, special attention was paid to the design of the screw drive, the screw thread, and the screw dimensions and shape. Several biomechanical tests were performed, including measurements of the insertion torque during graft fixation, ultimate failure load of the graft fixation, and ultimate failure torque of the screw.

The observed mean insertion torque of the interference screw during graft fixation was 1125±369.58 N mm and, therefore, slightly higher than the insertion torque of 822 ± 289 N mm reported in a recent study analyzing the biomechanical stability of the allograft interference screw in human proximal tibia specimens [40]. This was to be expected, since the insertion torques in bovine bone are generally higher than in human bone [41].

Compared to the ultimate failure torque of bioabsorbable interference screws, the allograft interference screw developed here is in the mid-range at 2633.33±152.75 N mm [41]. This speaks to the strength of the selected screw drive, and shows that the allograft screw is as stable as a commercial bioabsorbable screw in terms of torsional strength. The ultimate failure torque is more than twice the maximum occurring insertion torque. Therefore, there is sufficient safety that under normal circumstances there should be no failure of the screw due to excessive torsional loading during fixation of the graft.

The FEA in Figure 4c showed the highest von Mises stress in the area at the bottom of the external hexagon screw drive at about 2500 N mm. The measurements of the ultimate failure torque showed that the screws fail also at the bottom of the external hexagonal screw drive at about 2600 N mm. It can therefore be concluded that the FEA allows reliable assertions to be made about the strength of the screw drives investigated. For simplicity, a linear FEA with isotropic material properties was used. As cortical bone is basically a non-linear orthotropic material, a non-linear, orthotropic FEA would probably provide more precise results. However, this would lead to a much more complex problem requiring high processing power. In addition, Autodesk Fusion 360 software does not support othotropic materials in FEA applications. Since the problem at hand is a contact problem, singularities occur at the contacts between the screw and the insertion tool, as well as at sharp edges. The stresses occurring at these points are subsequently unrealistically high, and can therefore be neglected. In this case, the decisive factor is how far the areas with high stresses are distributed over the component; the further these areas extend over the component, the more likely material failure is. Thus, it is primarily the distribution of stresses that is critical to an assessment of strength and stability.

The measured mean ultimate failure load of the graft fixation with the allograft interference screw was 235.08±88.54 N, and thus lower than the ultimate failure loads of bioabsorbable screws with similar dimensions reported in other studies. For example, Herrera et al. found a mean load to failure of 9×23 screws (diameter × length in mm) of 294.44 N in a porcine model (screw dimension of the allograft interference screw developed here 8×21), and even higher loads for larger screw dimensions [42]. Therefore, it must be emphasized that the allograft screw tested here is with a diameter of 8 mm, slightly smaller compared with the screw in [42], and therefore the lower ultimate failure load is reasonable. As mentioned above, the reason for limiting the length of the allograft interference screw is due to the dimensions of the cortex (thickness of the cortex and length over which there is sufficient thickness). So, one could conclude that larger thread length of interference screws lead to higher ultimate failure loads, as was reported in previous studies [43]. Nevertheless, there are also studies that do not show a significant difference in ultimate failure load as a function of screw length [43]. These different results can possibly be explained by different approaches and surgical techniques, as well as different measurement setups, and may require more detailed investigations.

Weiss et al. performed a biomechanical analysis of ACL graft fixation in porcine and bovine models, respectively, using, among other methods, metallic interference screws with comparable dimensions (9×30 mm) [44]. In the interference screw group, a load to failure of about 270 N was observed, which is in the range of results obtained by [42] and by the allograft interference screw tested here. Another recent study by Suryavanshi et al. addressed the development of an interference screw using a novel composite biomaterial [45]. The results of biomechanical analysis using a synthetic bone block showed a pull-out force of about 325 N (7×25 mm screw). For comparison, they also tested a marketed bioabsorbable screw made of polylactide (PLLA) (8×20 mm), which had a pullout strength of about 250 N. Again, both results are basically in the same range as the allograft screw investigated in this work. In contrast, however, there is also literature reporting considerably higher ultimate failure loads when graft fixation is performed with interference screws. Schumacher et al. were able to determine the pull-out force of a novel hydroxyapatite-based screw to be approximately 400 N in sheep bone and approximately 490 N in polyurethane (PU) foam, respectively [21]. Moreover, Dong et al. recently reported a mean pull-out force of a biocomposite interference screw of about 460 N [46]. From the literature available so far, it can be concluded that the determined ultimate failure loads of different interference screws vary sometimes, significantly and should therefore rather not be used as the only criteria for the quality of graft fixation. Nevertheless, the allograft screw developed here is in a similar range, albeit somewhat lower in some cases.

Compared to the previous study analyzing the biomechanical stability of the allograft interference screw in human proximal tibia specimens [40], the ultimate failure load measured here is higher (235.08±88.54 N compared to 174.9±82.9 N). Again, this was to be expected, as bovine or porcine models tend to over estimate ultimate failure load of the graft fixation, hence the results achieved of in vitro animal models cannot be directly transferred to a clinical setting [47]. At this point, it should be mentioned that the bone mineral density (BMD) of the cancellous bone blocks in which the grafts were fixed was not determined, as there are no facilities for this. The BMD may vary depending on the selected bone block. This, of course, affects the stability of the graft fixation, as well as the insertion torque, and may influence the measurement results accordingly [48].

Of course, the selected transplant itself can also influence the strength of the fixation. Different folding and suturing techniques may well have an influence on the ultimate failure load of the graft fixation and the insertion torque of the screw [49]. For the sake of simplicity, only the simple single-fold technique was used in this work. Moreover, there are studies that show an influence of the graft length on the stress–strain pattern within the graft [50]. However, this parameter was not investigated in detail in the course of this work.

Furthermore, in agreement with previous publications [51], a significant correlation between insertion torque during graft fixation and ultimate failure load of the graft fixation could be observed (p=0.024). Of course, the insertion torque and thus also the ultimate failure load depend to a certain extent on the tunnel diameter or the ratio of the tunnel diameter to the graft thickness and the screw diameter. In other words, the smaller the tunnel diameter compared to the graft diameter and the screw diameter, the higher the insertion torque, and thus also the ultimate failure load. However, this also means that the screw is more difficult to insert, and the graft may rotate or even be damaged during insertion. In this work, the tunnel diameter was increased by 0.5 mm or 1 mm larger than the graft diameter, as this ratio enabled good screw insertion. Variation of the tunnel diameter was not performed in the course of this work, and would possibly be interesting for further research.

The sample size of the 9 mm graft group is n=4, only half the size of the 7 mm graft group. This is due to the fact that only a few tendons, which were thick enough for the 9 mm group, were available. The sample size of the ultimate failure torque test is rather small, n=3, which possibly influences the significance of the statistics. The reason for this small sample size is that the allograft screws are rather precious and expensive. Since the screws have to be destroyed for testing the ultimate failure torque, the sample size was chosen to be as small as necessary.

Looking at early functional rehabilitation programs after ACL injuries, in which loads of approximately 450 N have been reported [52], the primary stability of about 235 Nmm of the graft fixation with the allograft interference screw observed here is below this target value. However, the main advantage in terms of secondary stability of the allograft screws should now be considered. Since allograft screws from surgebright GmbH are typically freeze-dried, i.e., the water content is reduced to a minimum for long-term storage at room temperature [53], rehydration of the allograft screw after the surgical procedure leads to significant swelling in a short time. The screw diameter is thus slightly increased, which should lead to an increase in compression on the graft, and thus possibly to an increase in the stability of the fixation. In addition, the stability of the fixation may be further enhanced by the onset of the bone remodeling process [26,27,28], and the resulting ingrowth of the graft over time. However, these effects could not be investigated with the here used bovine model; therefore, further research in this area will be needed in the future.

Here, it is especially important and interesting to analyze the surrounding tissue of the graft fixation in more detail. In a study, Tecklenburg et al. were able to show the differences in the healing process of ACL reconstruction between bioabsorbable and allograft interference screws over a period of two years [54]. For the two bioabsorbable screws tested in that study, significant degradation of the screws was observed after 24 months. However, no new bone formation was observed, leaving a cavity in which the bioabsorbable screw was originally located. In contrast, bony incorporation of the screw was observed within 24 months in the allograft interference screws studied there [54]. Conclusions regarding ingrowth and subsequent stable anchorage of the graft can therefore be drawn from this study. Furthermore, the allograft screw would offer the advantage that the graft is in contact with living tissue from all sides, and can therefore be appropriately supplied and revitalized. With conventional bioabsorbable (or even metallic) screws, the graft is only partially in contact with living tissue.

In addition to ACL reconstruction, another interesting field of application for the allograft interference screw is ACL revision. As ACL reconstruction is a very successful operation, revision rates range from 3% to 25% [55]. The risk for ACL revision is influenced by various parameters like graft fixation, operation technique, tunnel placement, graft selection, etc. [56]. In certain cases (e.g., in case of substantial tunnel-widening), a two-stage revision may be necessary [57]. The two-stage revision involves an initial bone-grafting-procedure in order to fill widened or misplaced tunnels, followed by sufficient time to allow healing of the bone graft [58]. The second stage is again an ACL reconstruction. Due to the bone remodeling process and, therefore, the full conversion of the allograft interference screw into autologous bone [26,27,28], the allograft interference screw could, in some cases, make a two-stage surgery unnecessary. The screw could be used to fill the bone defect and fix the transplant at the same time.

## 5. Conclusions

This work is intended to serve as an initial starting point for more detailed future investigations, and to provide a first glimpse of the performance of an allograft interference screw for ACL reconstruction. The biomechanical tests of the allograft interference screw developed in this work showed good results in the first step, compared to conventional interference screws. In terms of ultimate failure torque, the allograft screw is in the mid-range among bioabsorbable screws. However, the primary stability of the graft fixation with the allograft interference screw (235 Nmm at 8 mm screw diameter) is lower than that of bioabsorbable screws with similar dimensions (e.g., 294 Nmm at 9 mm screw diameter [42]). The intended benefit of allograft interference screws, i.e., increased secondary stability due to swelling and conversion into autologous bone, still has to be confirmed and investigated in detail in future studies after product clearance.

## Figures and Tables

**Table 1:** Mechanical material properties of the screws and the insertion tools used for the FEA (taken from [30]).

	Freeze-Dried Human Cortical Bone	Chromium Steel 1.4034
Density in g/cm^3^	1.022	7.7
Young’s modulus in GPa	5	215
Poisson’s ratio	0.36	0.3
Yield strength in MPa	108	650

**Table 2:** Results of the biomechanical tests regarding insertion torque, ultimate failure load, and ultimate failure torque.

Sample Number	Graft Diameter in mm	Tunnel Diameter in mm	Max. Insertion Torque in N mm	Ultimate Failure Load Graft Fixation in N	Ultimate Failure Torque in N mm
1	7	8	1600	203	2500
2	9	9.5	1200	281	2600
3	7	8	1500	312	2800
4	9	9.5	1600	362	-
5	7	8	900	160	-
6	7.5	8	400	91	-
7	7	8.5	1200	199	-
8	9	9.5	700	212	-
9	7	8	1000	359	-
10	7	7.5	900	114	-
11	7.5	8	1100	240	-
12	9	9.5	1400	288	-
Mean value 7 mm graft			1075	209.75	-
Standard deviation 7 mm graft			377.02	92.24	-
Min. value 7 mm graft			400	91	-
Max. value 7 mm graft			1600	359	-
Mean value 9 mm graft			1225	285.75	-
Standard deviation 9 mm graft			386.22	61.32	-
Min. value 9 mm graft			700	212	-
Max. value 9 mm graft			1600	362	-
Mean value overall			1125	235.08	2633.33
Standard deviation overall			369.58	88.54	152.75
Min. value overall			400	91	2500
Max. value overall			1600	362	2800

## Data Availability

The data presented in this study are included in the article.

## Abbreviations

The following abbreviations are used in this manuscript:

ACL	Anterior Cruciate Ligament
MRI	Magnetic Resonance Imaging
FEA	Finite Element Analysis
CAD	Computer Aided Design
BMD	Bone Mineral Density
PLLA	Polylactide
PU	Polyurethane

## References

[1] Gerami M., Haghi F., Pelarak F., Mousavibaygei S. (2022). Anterior cruciate ligament (ACL) injuries: A review on the newest reconstruction techniques. J. Fam. Med. Prim. Care.

[2] Rezende F.C., Moraes V.Y., Franciozi C.E., Debieux P., Luzo M.V., Belloti J.C. (2017). One-incision versus two-incision techniques for arthroscopically assisted anterior cruciate ligament reconstruction in adults. Cochrane Database Syst. Rev..

[3] Marieswaran M., Jain I., Garg B., Sharma V., Kalyanasundaram D. (2018). A Review on Biomechanics of Anterior Cruciate Ligament and Materials for Reconstruction. Appl. Bionics Biomech..

[4] Sanders T.L., Kremers H.M., Bryan A.J., Larson D.R., Dahm D.L., Levy B.A., Stuart M.J., Krych A.J. (2016). Incidence of Anterior Cruciate Ligament Tears and Reconstruction. Am. J. Sport. Med..

[5] Mall N.A., Chalmers P.N., Moric M., Tanaka M.J., Cole B.J., Bach B.R., Paletta G.A. (2014). Incidence and Trends of Anterior Cruciate Ligament Reconstruction in the United States. Am. J. Sport. Med..

[6] Lam M.H., Fong D.T., Yung P.S., Ho E.P., Chan W.Y., Chan K.M. (2009). Knee stability assessment on anterior cruciate ligament injury: Clinical and biomechanical approaches. BMC Sport. Sci. Med. Rehabil..

[7] Chhabra A., Starman J.S., Ferretti M., Vidal A.F., Zantop T., Fu F.H. (2006). Anatomic, Radiographic, Biomechanical, and Kinematic Evaluation of the Anterior Cruciate Ligament and Its Two Functional Bundles. J. Bone Jt. Surg..

[8] Lohmander L.S., Englund P.M., Dahl L.L., Roos E.M. (2007). The Long-term Consequence of Anterior Cruciate Ligament and Meniscus Injuries. Am. J. Sport. Med..

[9] Lansdown D.A., Xiao W., Zhang A.L., Allen C.R., Feeley B.T., Li X., Majumdar S., Ma C.B. (2020). Quantitative imaging of anterior cruciate ligament (ACL) graft demonstrates longitudinal compositional changes and relationships with clinical outcomes at 2 years after ACL reconstruction. J. Orthop. Res..

[10] Buckthorpe M. (2019). Optimising the Late-Stage Rehabilitation and Return-to-Sport Training and Testing Process After ACL Reconstruction. Sport. Med..

[11] Filbay S.R., Ackerman I.N., Russell T.G., Crossley K.M. (2017). Return to sport matters—longer-term quality of life after ACL reconstruction in people with knee difficulties. Scand. J. Med. Sci. Sport..

[12] Gokeler A., Dingenen B., Hewett T.E. (2022). Rehabilitation and Return to Sport Testing After Anterior Cruciate Ligament Reconstruction: Where Are We in 2022?. Arthrosc. Sport. Med. Rehabil..

[13] Zeng C., Lei G., Gao S., Luo W. (2013). Methods and devices for graft fixation in anterior cruciate ligament reconstruction. Cochrane Database Syst. Rev..

[14] Tibor L., Chan P.H., Funahashi T.T., Wyatt R., Maletis G.B., Inacio M.C.S. (2016). Surgical Technique Trends in Primary ACL Reconstruction from 2007 to 2014. JBJS.

[15] Paschos N.K., Howell S.M. (2016). Anterior cruciate ligament reconstruction: Principles of treatment. EFORT Open Rev..

[16] Zainal Abidin N.A., Abdul Wahab A.H., Abdul Rahim R.A., Abdul Kadir M.R., Ramlee M.H. (2021). Biomechanical analysis of three different types of fixators for anterior cruciate ligament reconstruction via finite element method: A patient-specific study. Med. Biol. Eng. Comput..

[17] Zainal Abidin N.A., Ramlee M.H., Ab Rashid A.M., Ng B.W., Gan H.S., Abdul Kadir M.R. (2022). Biomechanical effects of cross-pin’s diameter in reconstruction of anterior cruciate ligament—A specific case study via finite element analysis. Injury.

[18] Abidin N.A.Z., Kadir M.R.A., Ramlee M.H. (2020). Biomechanical effects of different lengths of cross-pins in anterior cruciate ligament reconstruction: A finite element analysis. J. Mech. Med. Biol..

[19] Athwal K.K., Lord B.R., Milner P.E., Gutteridge A., Williams A., Amis A.A. (2020). Redesigning Metal Interference Screws Can Improve Ease of Insertion While Maintaining Fixation of Soft-Tissue Anterior Cruciate Ligament Reconstruction Grafts. Arthrosc. Sport. Med. Rehabil..

[20] Siroros N., Merfort R., Liu Y., Praster M., Migliorini F., Maffulli N., Michalik R., Hildebrand F., Eschweiler J. (2023). Mechanical properties of a bioabsorbable magnesium interference screw for anterior cruciate ligament reconstruction in various testing bone materials. Sci. Rep..

[21] Schumacher T.C., Tushtev K., Wagner U., Becker C., große Holthaus M., Hein S.B., Haack J., Heiss C., Engelhardt M., El Khassawna T. (2017). A novel, hydroxyapatite-based screw-like device for anterior cruciate ligament (ACL) reconstructions. Knee.

[22] Luo Y., Zhang C., Wang J., Liu F., Chau K.W., Qin L., Wang J. (2021). Clinical translation and challenges of biodegradable magnesium-based interference screws in ACL reconstruction. Bioact. Mater..

[23] Xu B., Yin Y., Zhu Y., Yin Y., Fu W. (2021). Comparison of Bioabsorbable and Metallic Interference Screws for Graft Fixation During ACL Reconstruction: A Meta-analysis of Randomized Controlled Trials. Orthop. J. Sport. Med..

[24] Emond C.E., Woelber E.B., Kurd S.K., Ciccotti M.G., Cohen S.B. (2011). A Comparison of the Results of Anterior Cruciate Ligament Reconstruction Using Bioabsorbable Versus Metal Interference Screws. J. Bone Jt. Surg.-Am. Vol..

[25] Watson J.N., McQueen P., Kim W., Hutchinson M.R. (2015). Bioabsorbable interference screw failure in anterior cruciate ligament reconstruction: A case series and review of the literature. Knee.

[26] Pastl K., Schimetta W. (2021). The application of an allogeneic bone screw for osteosynthesis in hand and foot surgery: A case series. Arch. Orthop. Trauma Surg..

[27] Pastl K., Pastl E., Flöry D., Borchert G.H., Chraim M. (2022). Arthrodesis and Defect Bridging of the Upper Ankle Joint with Allograft Bone Chips and Allograft Cortical Bone Screws (Shark Screw^®^) after Removal of the Salto-Prosthesis in a Multimorbidity Patient: A Case Report. Life.

[28] Brcic I., Pastl K., Plank H., Igrec J., Schanda J.E., Pastl E., Werner M. (2021). Incorporation of an Allogenic Cortical Bone Graft Following Arthrodesis of the First Metatarsophalangeal Joint in a Patient with Hallux Rigidus. Life.

[29] Amann P., Pastl K., Neunteufel E., Bock P. (2022). Clinical and Radiologic Results of a Human Bone Graft Screw in Tarsometatarsal II/+III Arthrodesis. Foot Ankle Int..

[30] Lifka S., Baumgartner W. (2021). A Novel Screw Drive for Allogenic Headless Position Screws for Use in Osteosynthesis—A Finite-Element Analysis. Bioengineering.

[31] Bi Z. (2018). Chapter 12—Validation and Verification. Finite Element Analysis Applications: A Systematic and Practical Approach.

[32] Krone R., Schuster P. (2006). An Investigation on the Importance of Material Anisotropy in Finite-Element Modeling of the Human Femur. SAE Technical Paper.

[33] Park Y.S., Kwon H.B. (2013). Three-dimensional finite element analysis of implant-supported crown in fibula bone model. J. Adv. Prosthodont..

[34] Du W., Zhang J., Hu J. A Method to Determine Cortical Bone Thickness of Human Femur and Tibia Using Clinical CT Scans. Proceedings of the 2018 IRCOBI Conference Proceedings.

[35] Sadat-Ali M., Elshaboury E., Al-Omran A., Azam M.Q., Syed A., Gullenpet A. (2015). Tibial cortical thickness: A dependable tool for assessing osteoporosis in the absence of dual energy X-ray absorptiopmetry. Int. J. Appl. Basic Med. Res..

[36] Morris M., Williams J., Thake A., Lang Y., Brown J. (2004). Optimal screw diameter for interference fixation in a bone tunnel: A porcine model. Knee Surg. Sport. Traumatol. Arthrosc..

[37] Mlynarek R.A., Bedi A., Brown C.H. (2018). Intratunnel Anterior Cruciate Ligament Graft Fixation. The Anterior Cruciate Ligament.

[38] Ezechieli M., Ettinger M., König C., Weizbauer A., Helmecke P., Schavan R., Lucas A., Windhagen H., Becher C. (2016). Biomechanical characteristics of bioabsorbable magnesium-based (MgYREZr-alloy) interference screws with different threads. Knee Surg. Sport. Traumatol. Arthrosc..

[39] Mayr R., Heinrichs C.H., Eichinger M., Coppola C., Schmoelz W., Attal R. (2015). Biomechanical Comparison of 2 Anterior Cruciate Ligament Graft Preparation Techniques for Tibial Fixation: Adjustable-Length Loop Cortical Button or Interference Screw. Am. J. Sport. Med..

[40] Benca E., van Knegsel K.P., Zderic I., Caspar J., Strassl A., Hirtler L., Fuchssteiner C., Gueorguiev B., Windhager R., Widhalm H. (2022). Biomechanical evaluation of an allograft fixation system for ACL reconstruction. Front. Bioeng. Biotechnol..

[41] Costi J.J., Kelly A.J., Hearn T.C., Martin D.K. (2001). Comparison of Torsional Strengths of Bioabsorbable Screws for Anterior Cruciate Ligament Reconstruction. Am. J. Sport. Med..

[42] Herrera A., Martínez F., Iglesias D., Cegoñino J., Ibarz E., Gracia L. (2010). Fixation strength of biocomposite wedge interference screw in ACL reconstruction: Effect of screw length and tunnel/screw ratio. A controlled laboratory study. BMC Musculoskelet. Disord..

[43] Efe T., Bauer J., Herdrich S., Gotzen L., El-Zayat B.F., Schmitt J., Schofer M.D. (2010). Comparison between bovine bone and titanium interference screws for implant fixation in ACL reconstruction: A biomechanical study. Arch. Orthop. Trauma Surg..

[44] Weiss F.P., Possoli F.A.d.A., Costa I.Z., Borges P.C., Stieven E., Kubrusly L.F. (2019). Fixation of the Anterior Ligament Graft at the Tibial Pole: Biomechanical Analysis of Three Methods. Rev. Bras. Ortop..

[45] Suryavanshi A., Khanna K., Sindhu K.R., Bellare J., Srivastava R. (2019). Development of bone screw using novel biodegradable composite orthopedic biomaterial: From material design to in vitro biomechanical and in vivo biocompatibility evaluation. Biomed. Mater..

[46] Dong W., Huang X., Sun Y., Zhao S., Yin J., Chen L. (2021). Mechanical characteristics and in vitro degradation kinetics analysis of polylactic glycolic acid/*β*-tricalcium phosphate (PLGA/*β*-TCP) biocomposite interference screw. Polym. Degrad. Stab..

[47] Aga C., Rasmussen M.T., Smith S.D., Jansson K.S., LaPrade R.F., Engebretsen L., Wijdicks C.A. (2013). Biomechanical Comparison of Interference Screws and Combination Screw and Sheath Devices for Soft Tissue Anterior Cruciate Ligament Reconstruction on the Tibial Side. Am. J. Sport. Med..

[48] Brand J.C., Pienkowski D., Steenlage E., Hamilton D., Johnson D.L., Caborn D.N.M. (2000). Interference Screw Fixation Strength of a Quadrupled Hamstring Tendon Graft Is Directly Related to Bone Mineral Density and Insertion Torque. Am. J. Sport. Med..

[49] Prado M., Martín-Castilla B., Espejo-Reina A., Serrano-Fernández J.M., Pérez-Blanca A., Ezquerro F. (2013). Close-looped graft suturing improves mechanical properties of interference screw fixation in ACL reconstruction. Knee Surg. Sport. Traumatol. Arthrosc..

[50] Wan C., Hao Z., Li Z., Lin J. (2017). Finite element simulations of different hamstring tendon graft lengths and related fixations in anterior cruciate ligament reconstruction. Med. Biol. Eng. Comput..

[51] Nyland J., Kocabey Y., Caborn D.N. (2004). Insertion torque pullout strength relationship of soft tissue tendon graft tibia tunnel fixation with a bioabsorbable interference screw. Arthrosc. J. Arthrosc. Relat. Surg..

[52] Yang D.L., Cheon S.H., Oh C.W., Kyung H.S. (2014). A Comparison of the Fixation Strengths Provided by Different Intraosseous Tendon Lengths during Anterior Cruciate Ligament Reconstruction: A Biomechanical Study in a Porcine Tibial Model. Clin. Orthop. Surg..

[53] Ariffin A., Chan H., Yusof N., Mohd S., Ramalingam S., Ng W., Mansor A. (2019). Establishing Freeze Drying Process for Cortical and Cancellous Bone Allograft Cubes. J. Health Transl. Med. (JUMMEC).

[54] Tecklenburg K., Burkart P., Hoser C., Rieger M., Fink C. (2006). Prospective Evaluation of Patellar Tendon Graft Fixation in Anterior Cruciate Ligament Reconstruction Comparing Composite Bioabsorbable and Allograft Interference Screws. Arthrosc. J. Arthrosc. Relat. Surg..

[55] Wilde J., Bedi A., Altchek D.W. (2013). Revision Anterior Cruciate Ligament Reconstruction. Sport. Health Multidiscip. Approach.

[56] Persson A., Gifstad T., Lind M., Engebretsen L., Fjeldsgaard K., Drogset J.O., Forssblad M., Espehaug B., Kjellsen A.B., Fevang J.M. (2018). Graft fixation influences revision risk after ACL reconstruction with hamstring tendon autografts. Acta Orthop..

[57] Kim D.H., Bae K.C., Kim D.W., Choi B.C. (2019). Two-stage revision anterior cruciate ligament reconstruction. Knee Surg. Relat. Res..

[58] Noyes F.R., Barber-Westin S.D. (2006). Anterior Cruciate Ligament Revision Reconstruction. Am. J. Sport. Med..

